# Towards holistic insect monitoring: species discovery, description, identification and traits for all insects

**DOI:** 10.1098/rstb.2023.0120

**Published:** 2024-06-24

**Authors:** Rudolf Meier, Emily Hartop, Christian Pylatiuk, Amrita Srivathsan

**Affiliations:** ^1^ Center for Integrative Biodiversity Discovery, Museum für Naturkunde, Leibniz Institute for Evolution and Biodiversity Science, Invalidenstraße 43, 10115 Berlin, Germany; ^2^ Department of Natural History, NTNU University Museum, Norwegian University of Science and Technology, Trondheim, NO-7491, Norway; ^3^ Institute of Biology, Humboldt University, 10115 Berlin, Germany; ^4^ Institute for Automation and Applied Informatics, Karlsruhe Institute of Technology, Karlsruhe, Germany

**Keywords:** insect decline, megabarcoding, integrative taxonomy ecological traits, natural history

## Abstract

Holistic insect monitoring needs scalable techniques to overcome taxon biases, determine species abundances, and gather functional traits for all species. This requires that we address taxonomic impediments and the paucity of data on abundance, biomass and functional traits. We here outline how these data deficiencies could be addressed at scale. The workflow starts with large-scale barcoding (megabarcoding) of all specimens from mass samples obtained at biomonitoring sites. The barcodes are then used to group the specimens into molecular operational taxonomic units that are subsequently tested/validated as species with a second data source (e.g. morphology). New species are described using barcodes, images and short diagnoses, and abundance data are collected for both new and described species. The specimen images used for species discovery then become the raw material for training artificial intelligence identification algorithms and collecting trait data such as body size, biomass and feeding modes. Additional trait data can be obtained from vouchers by using genomic tools developed by molecular ecologists. Applying this pipeline to a few samples per site will lead to greatly improved insect monitoring regardless of whether the species composition of a sample is determined with images, metabarcoding or megabarcoding.

This article is part of the theme issue ‘Towards a toolkit for global insect biodiversity monitoring’.

## 1. Introduction

Insects deliver many ecosystem services such as pollination, nutrient recycling, seed dispersal, etc. They also transmit pathogens, prey on other species and are an important food source [1–3]. Given the high species diversity and biomass of insects, it is inconceivable that any ecosystem can be understood and monitored without comprehension of the ecology of its insect communities. This is why biomonitoring should not stop with determining species richness, but also resolve taxonomic issues and include an assessment of abundance and functional diversity [4,5]. However, how can we obtain all this information? We here outline how to go from mass samples collected with standardized traps (e.g. Malaise traps) to generating data for functional analysis. The workflow starts with individually barcoding all specimens in a mass sample (‘megabarcoding’ [6]), grouping them into molecular operational taxonomic units (MOTUs), revising the MOTU boundaries to obtain species limits, training artificial intelligence (AI) identification algorithms for common species, and using vouchers to collect trait and natural history data at scale. Once this pipeline has been applied to a few samples at each monitoring site, all subsequent monitoring becomes easier, because AI tools can be used to identify at least some of the common species. In addition, species lists and abundances become more meaningful, because the samples can be analysed at species-level and use trait data for functional analysis.

**Figure 1.**
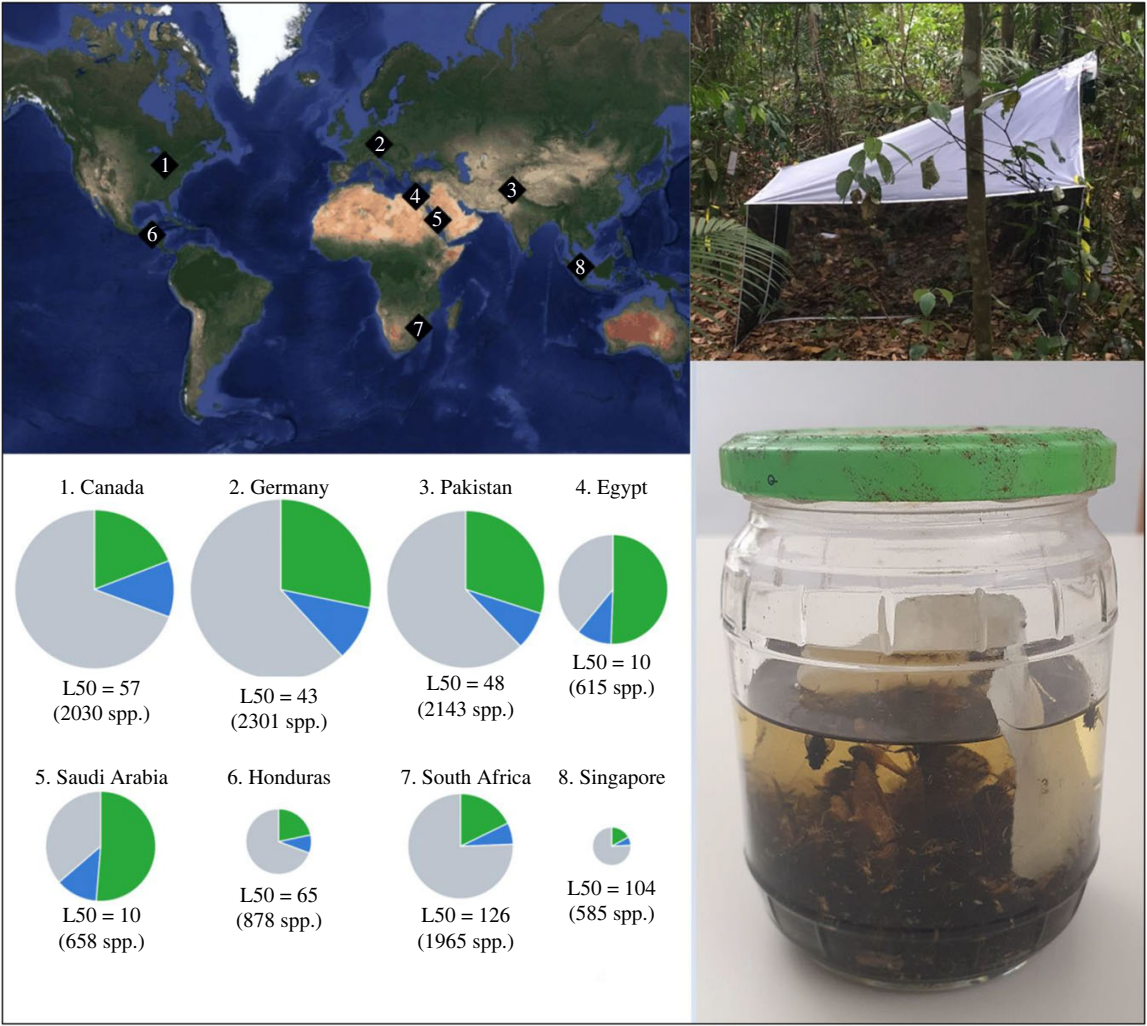
Proportion of specimens in Malaise traps belonging to the top 10 (green), next 10 (blue) and remaining (grey) MOTUs in Malaise traps characterised in Srivathsan *et al*. [9]. Pie charts are scaled to abundance. L50 values represent the number of MOTUs that cover 50% of the specimens. Map made using ggmap in R (Google maps, satellite, 2023 NASA).

Given the enormous number of insect species on our planet, the vision outlined above does come with many challenges. Before we outline the workflow in more depth, we would thus like to mitigate some typical concerns, while simultaneously stressing specific advantages. At present, only very few, charismatic species (e.g. some species of birds, butterflies, trees, etc.) are really monitored at a global scale [7]. By contrast, insect biomonitoring is conducted locally at a limited number of sites. Monitoring only becomes ‘global’ through the analysis of trends across local sites. Given this approach to global monitoring it is important that local sites are analysed in detail. This means that we need methods that go well beyond generating species lists. Such data should include abundance, trait, and natural history information for key species. Fortunately, gathering meaningful trait and natural history information for locally dominant species is made distinctly easier by typical patterns of species abundances [8]: most specimens at a site belong to a moderate number of species. These species then become a priority for collecting trait and natural history data. For example, analysis of Malaise trap data from eight countries in Srivathsan *et al*. [9] shows that on average, 39% of the specimens in a trap belong to the 20 most abundant species. Depending on sample, biogeographic region and habitat, the number of species contributing 50% of all specimens ranged from 7 to 195 (median = 53) (figure 1; electronic supplementary material). This suggests that many functional insights into insect communities can be gained by collecting trait data for a manageable number of species. However, this requires that biologists let go of their usual habit of studying only particular taxa and/or rare species. Instead, we propose that the focus should be on abundant species. Such a shift to abundance-based research priorities will be desirable not only for holistic insect biomonitoring, but also for addressing taxonomic impediments. Srivathsan *et al*. [9] also showed that more than half the specimens and species in Malaise trap samples belong to only 20 family level insect taxa [9]. Since most of these taxa are neglected by taxonomists [9], the setting of taxonomic priorities according to abundance will automatically shift attention to taxa that are currently understudied.

The need for the holistic study of bulk samples also becomes apparent when we consider current approaches. Morphospecies sorting and cherry-picking specimens from bulk samples have been largely replaced with bulk sequencing techniques using metabarcoding and metagenomics [10,11]. These techniques were developed to allow entire samples to be characterized at once and en masse [11–13]. However, all metabarcoding comes with the downside of losing the association between DNA barcode and specimen. In addition, many popular metabarcoding techniques are destructive, requiring the homogenization of samples into ‘insect soups’ before sequencing, leaving no voucher specimens to validate results [14,19]. This is problematic, as the barcode region targeted by metabarcoding is only a few hundred base pairs of DNA long. For 10–20% of taxa, this information has repeatedly been shown to be insufficient for delimiting species [15,16]. This problem also remains when non-destructive methods for metabarcoding are used (e.g. mild lysis, extraction of DNA from preservatives [17,18]). A further complication of metabarcoding is that different DNA extraction methods and the use of different primers yield different results [18,19]. Thus, method development is still ongoing to ensure the comparability of the presence–absence and approximate abundance data obtained from bulk sequencing of whole samples across studies [11,17,20].

## 2. Towards holistic insect monitoring

Each insect sample is like a library documenting the environmental conditions in the habitat where the sample was collected. To adequately extract this information, we must resolve species limits and gather data on the natural history of these species. We thus argue that metabarcoding should be complemented with techniques that generate barcode databases for validated species, reveal how common these species are, and how they interact with the environment. The data obtained from such a workflow will increase the value of species lists regardless of whether they were collected in the past or will be collected in the future.

Our proposal is that each site chosen for biomonitoring should be covered by an in-depth analysis of a few samples obtained with the kind of traps that will also be used for routine biomonitoring. These samples should be analysed by applying megabarcoding [6] combined with semi-automatic imaging of all specimens. In addition to establishing accessible vouchers, such an approach will reveal species richness and abundances. The resulting vouchers are then available for collecting important trait data. For example, data obtained with genome skimming or low-coverage genome sequencing can be mined for ecological information (see below), but also used to construct phylogenetic relationships [21], determine genetic diversity [22], population connectivity [23] and population size [24] (for further reading, see Theissinger *et al*. [25]). Inferences about the environmental history of a monitoring site will thus cover long time periods ranging from the condition at the time of collecting over recent years (e.g. population connectivity [23]) to thousands of years ago (e.g. community assembly [26]) This information can then also enrich the analysis of samples processed with metabarcoding, because the trait data would be associated with specific species names and the species would be identifiable via barcodes. As outlined below, this is eminently feasible, because we already know how to get barcodes and images at scale and metagenomics or whole genome sequencing can yield much of the remaining information [27].

## 3. From megabarcoding to species

**Figure 2.**
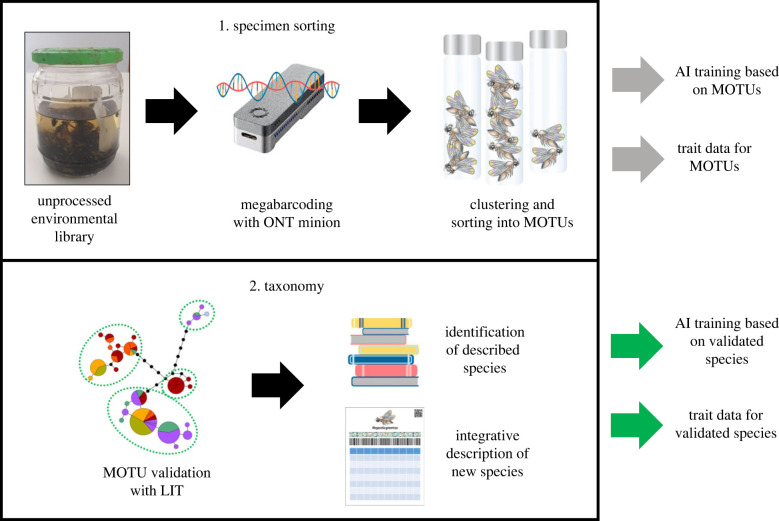
From bulk insect sample to species. Sorting involves megabarcoding and sorting specimens into MOTUs (ONT, Oxford Nanopore Technologies). Taxonomic research includes MOTU validation with LIT followed by either identification or description. AI training and trait data should be collected for validated species, but processing at MOTU-level can yield approximate data.

Bulk insect samples generally consist of a relatively small number of species in high abundance and a very large number of rare species (figures 1 and 2). Many abundant species belong to ‘dark taxa’, which Hartop *et al*. [28] defined as high-diversity clades (greater than 1000 species) that are poorly known (fewer than 10% of species described) [28]. What makes such taxa understudied is a combination of high abundance and diversity, usually accompanied by small size, poorly developed taxonomy and a reliance on microscopic characters for species identification [28,29]. Dark taxa are arguably the biggest obstacle for a holistic monitoring of insect communities. They are the main reason why, until recently, Malaise or pitfall trap samples were very rarely fully processed. Such dark taxa can be resolved at scale when bulk samples are processed by megabarcoding (figure 2; [6,9,30–33]). Such individual barcoding of all specimens is now feasible owing to the plummeting cost, high efficiency and rapidly increasing accuracy of third generation sequencing [34]. Megabarcoding associates every specimen in a sample with a DNA barcode and relies on use of non-destructive techniques to preserve morphological features of the specimens [31]. It thus generates a large number of vouchers that allow for downstream taxonomic and genomic research. One important use of these vouchers is species delimitation and description based on multiple types of data (integrative taxonomy) [35,36]. Recently, large-scale integrative taxonomy (LIT) was proposed as a systematic way to generate integrative species hypotheses [28]. Preliminary hypotheses are generated based on a data source such as DNA barcodes that is inexpensive and easy to obtain at scale. The hypotheses are then validated or revised using a second type of data obtained for a smaller number of specimens that was specifically chosen to test the preliminary hypotheses (e.g. based on haplotype divergence [28]).

LIT has been successfully tested twice. The first test [28] was based on approximately 18 000 specimens shown to contain 365 species of Phoridae (Diptera), although the specimens were initially only assigned to 315 molecular clusters. Barcodes were obtained for all specimens, but the targeted morphological checks only required revisiting just over 5% of specimens. A second successful validation of LIT was recently undertaken by Meier *et al*. [37] for 1456 specimens of Mycetophilidae (Diptera) which were shown to contain 120 species based on a LIT analysis. Compared to the first test based on phorid flies, a larger proportion of specimens had to be checked for MOTU validation because studying only one specimen for each of the 120 species already meant inspecting greater than 8% of all specimens in the sample (*n* = 1456). Still, LIT was again found to be effective at resolving conflict between MOTUs and morphology by studying the morphology of only a small subset of all specimens [37,38].

In the above applications of LIT, DNA barcodes were the data source used to generate preliminary species hypotheses and morphology was the second data source used to validate these hypotheses. However, LIT could also include other types of data. For example, automated imaging combined with AI algorithms for unsupervised learning may eventually advance sufficiently for new solutions: the first data source could be image data, while the validation of preliminary species hypothesis could be barcodes or morphological data from genitalia. Since automated imaging could yield thousands of images at low cost, this would be in line with the basic principle of LIT, i.e. that the data used for sorting should be cheap.

Delimiting all species found in a sample precedes one of the most difficult work phases in biodiversity science—identification or description. The task of distinguishing between species that have already been described and those that need description is often complicated by crumbling type material, muddled public databases, and insufficiently detailed legacy descriptions in the historical literature [36,39]. Resolving legacy issues in taxonomy is time-consuming, but this time only needs to be invested once per taxon and region. For example, once the described species of fungus gnats (Diptera: Mycetophilidae) in Southeast Asia had been evaluated based on descriptions and types, Amorim *et al*. [38] were able to describe 115 of the 120 species of one set of Malaise trap samples from Singapore as new to science. When Meier *et al*., analysed a second set of samples, more than 90% of the specimens belonged to the species described based on the first set samples. Indeed, the barcodes suggested the presence of fewer than 20 additional new species [37]. This implies that a few iterations of taxonomic revision of a taxon at any one site will be sufficient for allowing most species to be assigned to a species. The use of barcode and morphological data will also mean that the species are robustly delimited and can be identified with either barcodes or morphological features [28,38,40]. Associated high quality images will enable future-proofing the descriptions.

## 4. Robotics, machine learning and biomass

Processing several bulk samples per site using specimen-based approaches requires the use of robotics. In fact, we argue that the ultimate goal should be to develop AI identification algorithms, so that specimens can be largely identified based on images. Robots such as the DiversityScanner [41] have already been deployed to accelerate specimen transfer from bulk samples to 96-well microplates. This robot detects, photographs and sorts insects in preparation for megabarcoding [41]. Concomitant with the automation of barcoding, the DiversityScanner is programmed to generate stacked images for each specimen. These images (and associated specimens) are then grouped into putative species using barcode data. Even now, the DiversityScanner can already identify common families without sequencing, because it uses a trained convolutional neural network (CNN) for the most abundant insect families [41]. The next logical step is to train identification algorithms to recognize common species, first using MOTUs as approximations of species and later species after validation with LIT. Common species will benefit first, but even species that are rare in individual samples can eventually be covered by CNNs because specimens for rare species from many samples can be pooled until there are a sufficiently large number of images for training the algorithms.

At present, the extent to which CNNs will be able to identify specimens to species remains unclear [42]. However, this is probably a matter of what kinds of images (e.g. orientation, body part) are provided for training and identification. Importantly, the quality, variety, and orientation of images needed for CNN training may vary across taxa (see approaches used for nematodes: [43]). However, it is likely that the need for barcoding of specimens belonging to common species will dramatically decrease. Instead, such species will be identified based on images, then counted, and finally transferred into species-specific vials. Only unknown specimens will subsequently have to be picked up, imaged, and barcoded. This yields a continuous feedback loop that slowly whittles away the unknown biodiversity, and facilitates finding rare species that require additional sources of data, or expert attention. DNA barcoding may eventually be used only to validate identification generated by CNNs or for the identification of body parts that have lost diagnosable features.

The next step in holistic biomonitoring is semi-automatic collection of biomass information. Some tools are already available, but their use is still limited, because they either require identified specimens [44] or the biomass of three-dimensional insects is estimated based on two-dimensional images of whole samples [45] or individual specimens [41]. It appears to us that the next logical step is determining biomass based on three-dimensional models. They could initially be produced for common species by modelling several specimens covering the species' size range. Afterwards, one could establish the relationship between three-dimensional volumes and two-dimensional measurements so that routine biomonitoring could continue to rely on two-dimensional imaging. Once AI identification tools are available and conversion factors are known, one could spread an insect sample across a large table-sized tray and use cameras to identify and measure all specimens in almost real-time. When combined with information on what the species does in its environment, this will allow us to resolve changes in not only the size distribution and abundance of insects over time, but also to relate such changes to ecosystem stability. Such advances will get us one step closer to reading a bulk insect sample like we would read the books in an environmental library.

## 5. Large-scale natural history data

Holistic insect biomonitoring requires an understanding of the functional diversity of insect communities detected at particular sites [46,47]. Characterization of functional diversity ranges from using classifications of species into functional groups to the quantification of functional traits for species [48–50]. Some trait information can be obtained from the natural history literature [51], but this literature is in decline [52] and tends to cover mostly charismatic species. We thus argue that collecting trait data has to be greatly expanded and more targeted on species that are particularly common and/or contribute much of the biomass in different trophic groups. Semi-automatic imaging [41] immediately provides information on morphological traits such as body size, biomass, hairiness, eye number and size [4]. The vouchers generated by megabarcoding can also be used to obtain other life-history traits such sex ratios, time of reproduction, egg or clutch size. Beyond morphological traits, metagenomics (via whole genome sequencing) of vouchers will arguably offer the most promising approach for collecting additional ecological insights. This technique can simultaneously detect many species interactions across a broad range of taxa without amplification-related biases [53]. Indeed, metagenomics is particularly suitable when there is a need to rapidly characterize the feeding ecology, host genetics, parasites and microbes of a particular species [54]. Note that the same low-coverage genomic data also enables reconstructing phylogenetic relationships and estimating the genetic diversity within species [27,55].

However, metagenomics remains expensive and metabarcoding will retain much of its importance. For example, prey detection via metabarcoding of the gut content of predators using targeted primers is now well established [56,57], as is the sequencing of pollen found on insects [58–60]. Indeed, pollen can sometimes simply be shaken off insects and used directly as DNA template [58–60]. Given that much of the biomass of holometabolous insects is accumulated by larvae, dietary metabarcoding is particularly needed for immature stages [61]. This requires that the larvae and adults are first associated via megabarcoding [62]. Even tools that were initially developed only for vertebrate detection via invertebrate derived DNA (iDNA) can now also be used for revealing species interactions between insects and vertebrates [63–67], and plants [68]. The study of iDNA is particularly scalable and cost-effective when applied to insect faeces or regurgitates, as neither substrate requires DNA extraction [69]. Other cost-effective techniques are the analysis of fresh or archived plants for arthropod DNA [70,71] and the analysis of spider webs for prey DNA [72,73]. Occasionally, the interpretation of the signal can be difficult, but tools such as metabolite screens can even resolve whether the detected iDNA originated from carrion or dung [74,75].

For monitoring environmental health and ecosystem functioning, the study of microbiomes [76,77] is becoming integral to understanding of insect ecology [78], but the analysis of microbiomes also allows for the detection of antimicrobial resistance among the microorganisms in a habitat and sometimes it can even resolve whether the resistance genes were acquired by the insects when feeding on contaminated food (as has been shown for cockroaches, houseflies, ants, mosquitoes: [79]). Unfortunately, it is currently unknown to what extent bacteria with such resistance genes are prevalent in common insects, but in-depth study of vouchers for common species can answer these questions. Note, that screens of host genomes and microbiomes are also important for discovering new insect-derived antimicrobial substances [80] and can reveal mutations related to pesticide resistance, thus predicting the susceptibility of insect populations and contributing to selecting appropriate control measures [81–83].

## 6. Conclusion

To achieve holistic insect monitoring, we must overcome taxon biases and the lack of trait data for common species. This can be done by paying more attention to resolving species limits and making species identifiable through a variety of user-friendly tools. We predict that image-based identification will eventually turn out to be the tool of choice for many species. In the meantime, DNA barcoding using third generation sequencing is a viable and valuable intermediate solution, because it also yields the barcodes needed for the analysis of environmental DNA with metabarcoding and metagenomics. Following megabarcoding, the next logical step in holistic biomonitoring is collecting functional traits at scale. Body size, biomass, feeding habits, seasonality, clutch and egg size, and many other traits are low-hanging fruits, but many additional traits can be obtained through shallow sequencing of whole vouchers or body parts. By using these methods for characterizing the most common species in a few samples per site, a transformative shift in the quality of insect monitoring will be feasible.

## Data Availability

The data used for this submission is available from Nature: https://www.nature.com/articles/s41559-023-02066-0 [84]. Supplementary material is available online [85].
